# Supplementary material for: The lifetime accumulation of multimorbidity and its influence on dementia risk: a UK Biobank study
Source: Brain Commun. 2025 Jul 16;7(4):fcaf222. doi: 10.1093/braincomms/fcaf222 (PMC12266833; doi:10.1093/braincomms/fcaf222)

Supplementary Materials

Supplementary Table 1: A list of the 46 chronic conditions assessed, the ICD codes used to derive diagnosis, and the abbreviated display name used for each condition in main text figures.

Condition	ICD10 Codes	Display Name
anaemia	D50,D51,D52,D53,D60,D61,D62,D63,D64,D55,D56,D57,D58,D59	Anaemia
anxietydisorders	F40,F41,F42	Anxiety
arthritis	M00,M01,M02,M03,M05,M06,M07,M08,M09,M13,M11,M14,M12,M15,M16,M17,M18,M19,M10	Arthritis
asthma	J45	Asthma
atherosclerosis	I70	Athero
atrial fibrillation	I48,I49	AF
bronchiectasis	J47	Bronch
cancer	C00,C01,C02,C03,C04,C05,C06,C15,C16,C17,C18,C19,C20,C21,C22,C25,C34,C40,C41,C43,C50,C53,C55,C56,C61,C64,C71,C80,C81,C82,C83,C84,C85,C86,C88,C90,C91,C92,C93,C94,C95,C96,C07,C08,C09,C10,C11,C12,C13,C14,C23,C24,C26,C30,C31,C32,C33,C37,C38,C39,C44,C45,C46,C47,C48,C49,C51,C52,C54,C55,C57,C58,C60,C62,C63,C65,C66,C67,C68,C69,C70,C72,C73,C74,C75,C76,C77,C78,C79,C97	Cancer
cataract	H25,H26	Cataract
chronic fatigue syndrome	R53	CFS
chronic kidney disorder	N18,N19,I120,I131,I132,E102,E112,E132	CKD
chronic obstructive pulmonary disorder	J44,J43,J42,J41,J40	COPD
chronicsinusitis	J32	Sinusitis
coronary heart disease	I20,I21,I22,I23,I24,I25	CHD
diabetes	E11,E10,E13,O24,E14,E12	Diabetes
depression	F32,F33,F204,F251,F412,F920	Deprn
diverticulardisease	K57,K314	Div
endometriosis	N80	Endomet

epilepsy	G40,F803	Epilepsy
glaucoma	H40,H42	Glaucoma
Gastro-oesophageal reflux disease	K21	GORD
hearingloss	H90,H91	Hearing
heartfailure	I50,I42,I130,I132	HF
hypertension	I10,I11,I12,I13,I15,I674	Hyt
irritable bowel syndrome	K58	IBS
inflammatory bowel disease	K50,K51,K52	IBD
liver disease	K70,K71,K72,K73,K74,K75,K76,K77	Liver
lipid disorder	E78	Lipids
macular degeneration	H353	MD
menieres	H810	Menieres
migraine	G43	Migraine
multiple sclerosis	G35	MS
obesity	E66	Obesity
osteoporosis	M80,M81,M82	Osteo
parkinsons disease	G20,G21	PD
peripheral vascular disease	I731,I738,I739,I730	PVD
prostate conditions	N40,N41,N42,N510	Prostate
psoriasis and eczema	L20,L21,L22,L23,L24,L25,L26,L27,L28,L29,L30,L40,L41	Psoriasis
psy	F20,F21,F22,F23,F24,F25,F28,F29,F30,F31,F323,F333	Psyc
pulmonary circulation disorder	I26,I27,I28	PCD
sleep disorders	F51,G47	Sleep
stress disorders	F43	Stress

stroke	I60,I61,I62,I63,I64	Stroke
transient ischaemic attack	G45	TIA
thyroid conditions	E00,E01,E02,E04,E05,E06,E07,E03	Thyroid
ulcer	K25,K26,K27,K28,K221	Ulcer

Supplementary Table 2: An illustration of our disease x subject matrix (stratified by age band) which was used as the input for the clustering analysis. As an illustrative example, we have presented the age at diagnosis for diabetes and hypertension, for four subjects. Within each age band (0-55, 55-65, 65-70), the diagnosis is binarised to reflect a novel diagnosis within that band, and those binarised results are the inputs for subsequent clustering.

	Diabetes				Hypertension				…
Subject No	Diagnosis Age	Age-band			Diagnosis Age	Age-band			
		0-55	55-65	65-70		0-55	55-65	65-70	
1	70	0	0	1	35	1	0	0	
2	NA	0	0	0	22	1	0	0	
3	40	1	0	0	NA	0	0	0	
4	25	1	0	0	68	0	0	1	
…									

Supplementary Table 3: Prevalence and observed/expected (OE) ratio of each chronic condition within each of five clusters defined from 0-55 years.

Condition	Cluster_1prevalence	Cluster_1OE	Cluster_2prevalence	Cluster_2OE	Cluster_3prevalence	Cluster_3OE	Cluster_4prevalence	Cluster_4OE	Cluster_5prevalence	Cluster_5OE
anaemia	1.3	0.68	4.5	2.39	3.7	1.95	3.1	1.66	6.7	3.53
AF	0.3	0.5	6.8	11.29	0.8	1.26	5.2	8.61	0.5	0.89
atherosclerosis	0	0	2.6	23.63	0.2	1.91	1.2	11.08	0	0.04
CHD	0	0	49.6	24.2	2.7	1.3	10.9	5.32	0.5	0.22
heartfailure	0	0	5.6	24.31	0.2	1.05	1.8	7.68	0	0.06
hypertension	3	0.56	43.7	8.16	14.5	2.71	31.6	5.9	9	1.68
PCD	0.2	0.75	1	3.97	0.4	1.49	1.5	5.67	0.5	1.83
PVD	0.2	0.48	2.9	6.59	0.9	2.14	2.1	4.8	1.5	3.45
stroke	0	0	0	0.1	0	0	69.5	191.83	0	0
TIA	0	0	0	0	0	0	35.8	192.58	0	0
diabetes	0.5	0.37	17.8	13	6.5	4.72	7.6	5.54	1.5	1.1
lipids	0	0	52.7	23.21	3.9	1.72	17.9	7.89	1.1	0.47
obesity	0.4	0.39	8.4	8.5	2.3	2.33	2.9	2.96	3.5	3.52
CKD	0	0.24	2.8	14.94	1	5.3	1.2	6.14	0.3	1.45
thy	0.8	0.63	4.6	3.65	2.8	2.26	2.9	2.27	4.3	3.4
liverdisease	0.2	0.58	1.6	5.76	0.7	2.61	1.2	4.15	0.8	2.78
diverticular disease	0.8	0.74	2.6	2.31	1.7	1.53	2	1.81	3.3	2.95
GORD	1.4	0.71	5.7	2.9	2.7	1.36	4.6	2.34	6	3.05
inflammatoryboweldisease	1.2	0.7	4.4	2.52	3.3	1.87	4.2	2.36	5.7	3.25
irritablebowelsyndrome	0	0	1.8	1.47	2	1.57	2.6	2.07	14	11.18
ulcer	1.4	0.79	4.5	2.62	2.6	1.49	4.8	2.8	3.9	2.25
hearingloss	0.8	0.65	2.3	1.85	2.6	2.14	2.7	2.16	5	4.06

menieres	0.1	0.75	0.3	2.03	0.1	0.87	0.5	3.92	0.4	3.02
cataract	0	0	0.4	0.49	60.4	83.04	1.4	1.87	0	0.02
glaucoma	0	0	0.1	0.17	36.6	83.82	1.2	2.81	0	0
maculardegeneration	0	0	0	0	8.2	84.99	0.1	1.42	0	0
endometriosis	0.4	0.84	0.5	1.08	0.5	1.15	0.8	1.82	1.2	2.59
prostateconditions	0.5	0.81	2	3.07	1	1.5	1.3	2	1.2	1.85
anxietydisorders	0	0	4.6	1.3	5.9	1.67	8.5	2.39	39.9	11.22
depression	0	0	5.2	1.42	6.5	1.8	9	2.47	40.6	11.14
stressdisorders	0	0	0.1	1.27	0.1	1.69	0.5	6.64	0.8	10.96
psy	0	0	0.3	1.12	0.3	0.96	0.5	1.92	3.2	11.44
PD	0	0.74	0	0.7	0.1	2.34	0.1	3.5	0.1	3.56
epilepsy	0.4	0.69	1.4	2.64	0.7	1.33	5.6	10.7	1.5	2.85
migraine	1.3	0.53	2.8	1.12	3.8	1.53	6.9	2.8	14.1	5.73
MS	0.2	0.74	0.4	1.61	0.3	1.37	0.8	3.7	0.7	3.19
sleepdisorders	0.3	0.71	2	4.39	0.9	1.99	1.3	2.84	1	2.18
CFS	0	0	1.5	1.56	1.7	1.67	4.5	4.52	10.9	10.96
arthritis	3.6	0.75	13.6	2.82	8.7	1.8	10.7	2.21	12.4	2.56
osteoporosis	0.4	0.77	1.1	1.95	1.2	2.21	1.2	2.24	1.5	2.75
asthma	2.1	0.73	8.9	3.02	6.9	2.35	6.2	2.1	7.9	2.67
bronchiectasis	0.1	0.82	0.2	1.49	0.4	2.48	0.5	3.25	0.3	2.3
chronicsinusitis	0.4	0.59	1	1.3	1.3	1.69	1.3	1.72	3.8	5.08
COPD	0.3	0.5	3	4.97	1.6	2.62	1.9	3.18	2.4	4
psoriasiseczema	1.5	0.6	3.8	1.53	4.4	1.78	5.7	2.32	11.9	4.82
cancer	2.7	0.9	4.1	1.35	5	1.63	4	1.3	5.4	1.77
dementia	2.5	1.04	2.4	0.96	1.5	0.61	2.5	1	1.7	0.7

Supplementary Table 4: Prevalence and observed/expected (OE) ratio of each chronic condition within each of five clusters defined from 55-65 years.

Condition	Cluster _1prevalence	Cluster _1OE	Cluster _2prevalence	Cluster _2OE	Cluster _3prevalence	Cluster _3OE	Cluster _4prevalence	Cluster _4OE	Cluster _5prevalence	Cluster _5OE
anaemia	1.6	0.47	10.2	3.06	5.6	1.67	6.8	2.04	11.2	3.35
AF	1	0.32	12.6	3.89	3.2	0.98	13.1	4.06	9.8	3.02
atherosclerosis	0	0	0	0	0	0	0.2	0.49	28.1	80.17
CHD	1.1	0.17	31.3	4.7	6.3	0.94	15.5	2.32	29.6	4.45
heartfailure	0.1	0.05	6	4.98	1.2	0.99	4.6	3.88	7.5	6.3
hypertension	8.8	0.48	59.4	3.22	23.7	1.29	45.6	2.47	41.3	2.24
PCD	0.3	0.47	2.2	3.1	1	1.43	2	2.71	2.3	3.2
PVD	0	0	0	0	0.1	0.12	1.6	1.55	78.7	78.18
stroke	0	0	0	0	0.2	0.21	64.4	57.24	3.5	3.12
TIA	0	0	0	0	0	0.03	41.8	59.09	1.1	1.5
diabetes	1.6	0.3	22.3	4.08	7.3	1.33	11	2.01	14.9	2.73
lipids	1.6	0.17	40.7	4.48	13.1	1.44	30.5	3.36	31	3.42
obesity	0.5	0.16	13.8	4.59	6.3	2.1	4.7	1.55	7.1	2.36
CKD	0.3	0.19	6.1	4.1	2.9	1.96	5	3.37	6.7	4.54
thy	2.1	0.56	10.3	2.72	7.2	1.89	5.8	1.52	6.2	1.64
liverdisease	0.4	0.38	4	3.45	2.4	2.05	2.2	1.91	3.3	2.79
diverticular disease	4.4	0.72	13.1	2.14	9.1	1.49	6.7	1.1	8.9	1.46
GORD	3.2	0.59	13.5	2.48	11.6	2.14	7.6	1.39	10.7	1.96
inflammatoryboweldisease	2.3	0.68	7.6	2.21	5.8	1.69	5.3	1.54	6.7	1.95
irritablebowelsyndrome	0.8	0.56	3	2.13	5.2	3.7	1.5	1.06	2.5	1.78
ulcer	1.3	0.55	6.9	2.87	3.4	1.4	4.3	1.81	5.5	2.31

hearingloss	2	0.78	3.8	1.51	5.7	2.26	3.9	1.55	3.9	1.57
menieres	0.1	0.64	0.5	2.39	0.4	1.76	0.3	1.56	0.3	1.26
cataract	3.5	0.79	7.9	1.79	6.4	1.44	5.4	1.21	7.9	1.78
glaucoma	1.2	0.83	2.2	1.57	2	1.4	2.2	1.6	2.1	1.49
maculardegeneration	0.4	0.71	1.1	2.05	0.9	1.59	0.7	1.3	1.4	2.46
endometriosis	0.2	0.87	0.3	1.41	0.4	1.62	0.2	0.78	0.3	1.31
prostateconditions	2.4	0.75	6.5	2.07	3.6	1.13	5	1.58	5.1	1.63
anxietydisorders	0	0	0.5	0.16	53.4	16.18	5.6	1.7	6.2	1.89
depression	0	0	1.4	0.37	59.7	15.48	7.6	1.98	8.2	2.13
stressdisorders	0	0	0	0	1.6	16.72	0.2	2.06	0.1	0.92
psy	0	0	0	0.01	5.1	16.76	0.4	1.27	0.5	1.71
PD	0.2	0.69	0.5	1.78	0.6	2.4	0.5	1.98	0.7	2.77
epilepsy	0.4	0.62	0.9	1.58	1.5	2.55	4.2	7.04	1.1	1.86
migraine	1.2	0.81	1.5	1.01	4	2.7	4.9	3.3	2.3	1.59
MS	0.1	0.91	0.2	1.24	0.2	1.24	0.2	1.04	0.4	2.43
sleepdisorders	0.5	0.43	3.5	2.97	3.1	2.65	2.4	2.06	2.7	2.28
CFS	0.6	0.61	1.5	1.6	2.9	3.07	4.8	5	2.2	2.34
arthritis	10.4	0.7	32.2	2.17	23.8	1.6	18.5	1.25	24.2	1.63
osteoporosis	2	0.83	3.5	1.41	4.7	1.9	3	1.22	4.6	1.88
asthma	3.9	0.62	15.8	2.53	10.6	1.7	8.8	1.4	10	1.6
bronchiectasis	0.3	0.54	1.5	2.84	0.9	1.79	0.7	1.45	1.1	2.22
chronicsinusitis	0.7	0.83	1.1	1.3	2	2.23	1	1.13	1.5	1.75
COPD	0.7	0.3	8	3.68	4.4	2.04	4.9	2.25	9.4	4.31
psoriasiseczema	2.2	0.77	4.6	1.59	6.4	2.2	4.4	1.5	5.1	1.76
cancer	7.8	0.88	13.5	1.53	10.3	1.16	8.7	0.98	9.7	1.1
dementia	2.3	0.93	2.9	1.19	2.8	1.14	4.2	1.7	3.5	1.45

Supplementary Table 5: Prevalence and observed/expected (OE) ratio of each chronic condition within each of five clusters defined from 65-70 years.

Condition	Cluster _1prevalence	Cluster _1OE	Cluster _2prevalence	Cluster _2OE	Cluster _3prevalence	Cluster _3OE	Cluster _4prevalence	Cluster _4OE	Cluster _5prevalence	Cluster _5OE
anaemia	10.8	3.51	5.8	1.87	11.1	3.59	0.8	0.27	6.7	2.17
AF	12	3.39	13.9	3.93	12.2	3.44	1	0.27	6	1.7
atherosclerosis	0	0	0.1	0.31	30.3	93.34	0	0	0	0
CHD	17.9	4	8.9	1.99	20.7	4.64	0.7	0.15	7.2	1.61
heartfailure	5.1	3.9	4.3	3.32	8.2	6.33	0.1	0.11	2.6	1.98
hypertension	46.5	3.42	31	2.28	28.1	2.07	4.5	0.33	20.5	1.51
PCD	2.5	3.65	2.4	3.47	2.5	3.67	0.1	0.2	1.4	2.08
PVD	0	0	0.7	0.85	75.6	91.2	0	0	0.3	0.36
stroke	0	0	57	51.27	3.5	3.12	0	0	0.6	0.57
TIA	0	0	30.8	53.14	0.7	1.12	0	0	0.1	0.2
diabetes	14.1	3.38	7.7	1.85	8.9	2.14	1.5	0.35	6.3	1.52
lipids	27.9	3.75	22.2	2.98	24.5	3.28	1.5	0.2	13.8	1.85
obesity	10.7	3.97	4.4	1.64	6.4	2.36	0.5	0.17	6.1	2.24
CKD	7.3	3.55	4.7	2.26	8.9	4.33	0.5	0.23	5	2.41
thy	9.6	3.44	4.3	1.55	5.6	2	0.9	0.32	5.9	2.11
liverdisease	3.4	3.62	1.5	1.6	2.9	3.01	0.2	0.24	2.4	2.53
diverticular disease	11.6	2.01	7.3	1.27	9.5	1.65	4.1	0.71	8.9	1.53
GORD	12.7	3.01	6	1.42	7.7	1.83	1.8	0.42	9.9	2.34
inflammatoryboweldisease	4.8	2.58	3.7	1.99	3.2	1.73	1	0.55	3	1.6
irritablebowelsyndrome	2.5	2.75	1.4	1.49	1.9	2.05	0.4	0.41	3.4	3.7
ulcer	5.1	3.51	2.1	1.44	3.1	2.13	0.5	0.32	2.5	1.74

hearinglos s	3.7	1.74	3.7	1.74	3.6	1.67	1.6	0.74	4.4	2.07
menieres	0.3	3.06	0.2	1.78	0.3	2.31	0	0.36	0.3	2.96
cataract	9.8	1.73	6.8	1.2	8.4	1.48	4.3	0.75	12.1	2.13
glaucoma	2.7	1.87	2.2	1.54	2.3	1.64	1	0.73	2.6	1.81
macularde generation	1.8	2	0.9	1.01	1.3	1.41	0.6	0.68	2.1	2.39
endometrio sis	0.3	3.32	0	0.27	0	0	0	0.41	0.2	2.09
prostateco nditions	7.9	2.79	4.6	1.63	4.8	1.68	1.5	0.52	3.9	1.36
anxietydiso rders	0	0.01	3	1.48	2.8	1.35	0	0	54.6	26.56
depression	0	0	4.2	1.95	4.7	2.21	0	0	55.9	26.08
stressdisor ders	0	0	0	0	0	0	0	0	1.1	27.76
psy	0	0	0.2	0.76	0.3	1.54	0	0	5.3	26.91
PD	0.5	1.7	0.6	1.85	0.5	1.54	0.2	0.71	0.9	2.93
epilepsy	0	0.01	19.2	51.11	0.7	1.97	0	0	0.4	0.96
migraine	0.9	1.43	3.1	4.9	1.2	1.9	0.5	0.71	1.7	2.59
MS	0.1	1.86	0.1	1.98	0.1	1.37	0	0.71	0.1	2.03
sleepdisor ders	2.4	3.06	1.6	2.02	2	2.61	0.3	0.33	2.6	3.27
CFS	1.4	2.29	2.6	4.44	1.6	2.78	0.3	0.48	1.8	2.99
arthritis	28.6	2.61	13.8	1.26	17.9	1.63	6	0.55	20.5	1.87
osteoporos is	4	1.89	3.2	1.49	4.8	2.24	1.5	0.7	4.6	2.15
asthma	11.7	2.86	6.6	1.61	7	1.72	1.9	0.48	7.6	1.85
bronchiect asis	1.8	3.23	0.8	1.42	1.3	2.22	0.2	0.37	1.2	2.13
chronicsinu sitis	0.7	1.65	0.7	1.61	0.6	1.39	0.3	0.75	1	2.33
COPD	8.6	3.59	5.8	2.42	9.2	3.86	0.5	0.22	5.7	2.37
psoriasisec zema	3.3	2.04	2.7	1.66	3.8	2.39	1.1	0.67	2.9	1.83
cancer	18.6	2.45	9.6	1.26	10.8	1.42	4.6	0.61	11.5	1.51
dementia	3	1.1	6.1	2.26	3.4	1.27	2.4	0.91	4.5	1.69

Supplementary Table 6: Association of multimorbidity clusters and incident dementia when assessing each timepoint individually. To first assess the cross-sectional association of each multimorbidity cluster, irrespective of prior or future multimorbidity, we analysed each age range independently and found all multimorbidity clusters were significantly associated with increased risk of incident dementia ($P_{FDR} < 0.05$) with the exception of the EYE cluster in the 0-55 age range. Age, sex, and years of education were covariates in all models.

**Multimorbidity patterns at 0-55 years**				
**Predictor**	**beta**	**Odds Ratio [95% CI]**	p	p_{BH}
Intercept	-17.09		0	0
CVMTB	0.61	1.84 [1.61, 2.1]	1.43E-19	2.86E-19
EYE	0.06	1.06 [0.78, 1.4]	0.69	0.69
MH	0.12	1.13 [1.02, 1.26]	0.02	0.03
NVASC	0.67	1.96 [1.37, 2.71]	9.33E-05	1.24E-04
Age	0.22	1.24 [1.24, 1.25]	0	0
Male Sex	0.16	1.18 [1.12, 1.24]	7.49E-11	1.20E-10
Education (years)	-0.05	0.95 [0.95, 0.96]	1.88E-27	5.02E-27
**Multimorbidity patterns at 55-65 years**				
**Predictor**	**beta**	**Odds Ratio [95% CI]**	p	p_{BH}
Intercept	-17.51		0	0
CVMTB	0.49	1.64 [1.53, 1.75]	2.06E-49	5.50E-49
MH	0.63	1.88 [1.7, 2.08]	2.83E-34	5.66E-34
NVASC	0.77	2.16 [1.85, 2.5]	3.10E-23	4.95E-23
PVASC	0.55	1.73 [1.43, 2.08]	8.97E-09	8.97E-09
Age	0.22	1.25 [1.24, 1.26]	0	0
Male Sex	0.15	1.16 [1.1, 1.22]	4.33E-09	4.95E-09
Education (years)	-0.04	0.96 [0.95, 0.97]	5.75E-22	7.67E-22

Multimorbidity patterns at 65-70 years				
**Predictor**	**beta**	**Odds Ratio [95% CI]**	p	p_{BH}
Intercept	-19.84		0	0
CVMTB	0.21	1.24 [1.16, 1.33]	5.21E-10	6.94E-10
MH	0.93	2.54 [2.26, 2.84]	2.66E-56	7.10E-56
NVASC	0.96	2.61 [2.27, 2.99]	8.87E-42	1.77E-41
PVASC	0.33	1.39 [1.09, 1.73]	0.01	0.01
Age	0.26	1.29 [1.28, 1.3]	0	0
Male Sex	0.14	1.15 [1.09, 1.22]	2.75E-07	3.14E-07
Education (years)	-0.04	0.96 [0.95, 0.97]	1.19E-20	1.91E-20

Supplementary Figure 1: Plot showing computed silhouette values for different values of k, for each of the multimorbidity age ranges in which clustering was performed. A high silhouette value indicates within cluster coherence. The 55-65 and 65-70 clusterings (performed with N=282,712 and N=216,372, respectively) decline until k=6, with a moderate levelling off after. The 0-55 clustering (N=282,712) shows moderate increase until k=8. Largely, for each age range, silhouette values were relatively consistent. We thus selected to analyse k=5 across age ranges, selecting the granularity prior to a dip in coherence is observed at 55-65 and 65-70. Assessing the same number of clusters across age ranges better enables us to track accumulation of repetitive multimorbidity patterns.

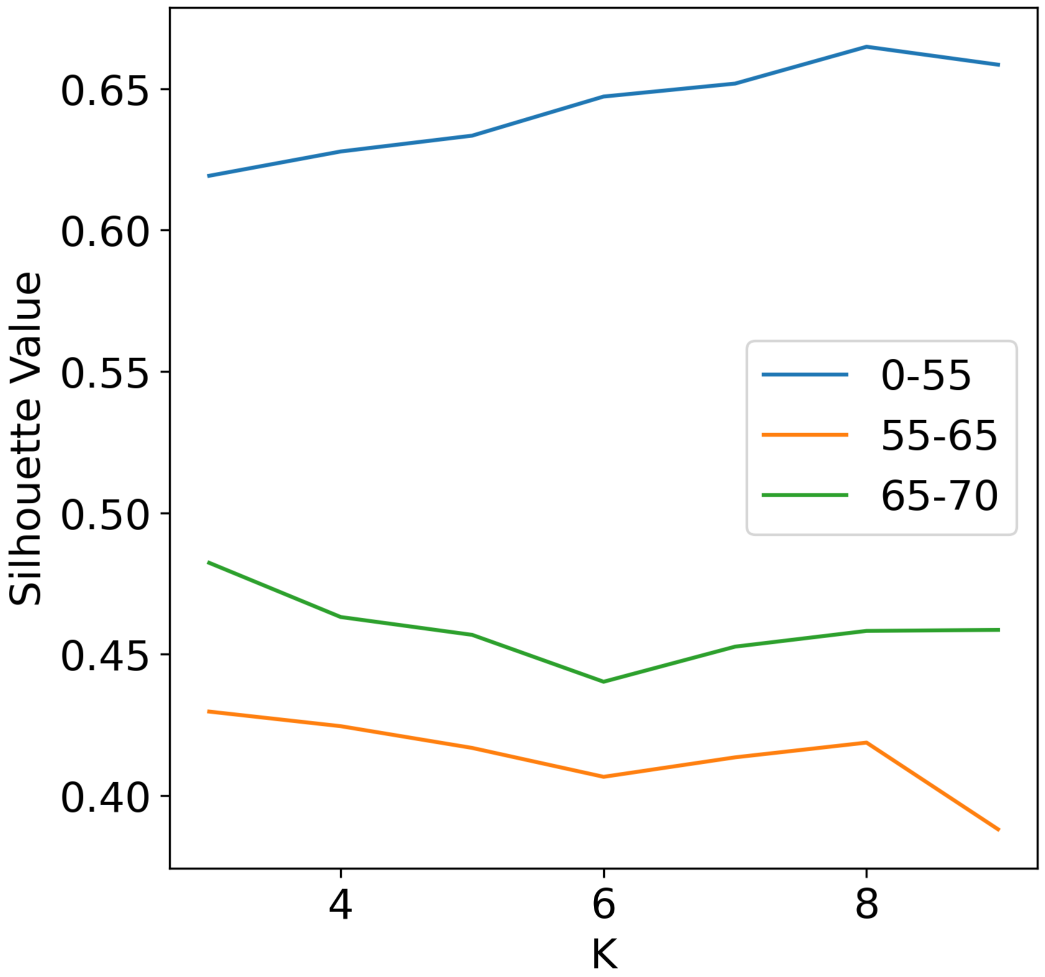

Supplementary Figure 2: Plot showing the odds ratios (ORs) for each multimorbidity cluster within each age band relative to the LOW cluster. Logistic regression was performed with incident dementia as outcome, multimorbidity cluster from 0-55, multimorbidity cluster from 55-65, and multimorbidity cluster from 65-70 as predictors, and age, sex, and years of education as covariates.

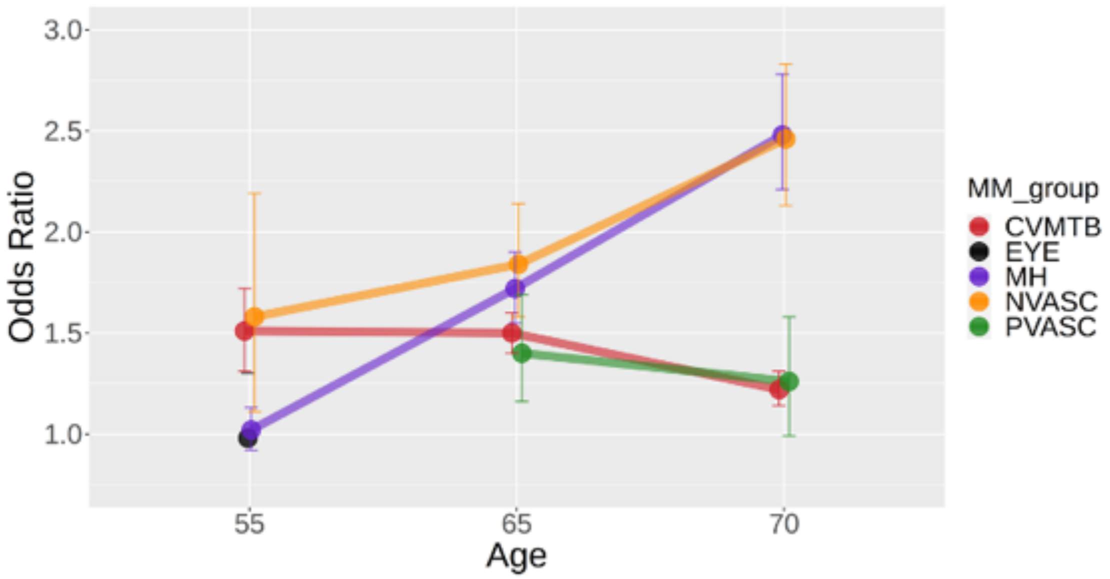

Supplement: fcaf222_Supplementary_Data [file fcaf222_supplementary_data.pdf]
